# Non-Verbal Cognitive Abilities in Children and Adolescents Affected by Migraine and Tension-Type Headache: An Observational Study Using the Leiter-3

**DOI:** 10.3389/fneur.2018.00078

**Published:** 2018-03-05

**Authors:** Lucia Margari, Roberto Palumbi, Paola A. Lecce, Francesco Craig, Marta Simone, Mariella Margari, Silvana M. C. Seccia, Maura Buttiglione

**Affiliations:** ^1^Basic Medical Sciences, Neuroscience and Sense Organs Department, University of the Study of Bari Aldo Moro, Azienda Ospedaliero-Universitaria Consorziale Policlinico di Bari, Bari, Italy; ^2^Scientific Institute Eugenio Medea (IRCCS) - La Nostra Famiglia, Brindisi, Italy

**Keywords:** migraine, tension-type headache, non-verbal cognitive abilities, non-verbal memory, attention skills, childhood, adolescence

## Abstract

Headache is one of the most common neurological disorders in developmental age. Several studies investigated the relationship between headache and emotional/behavioral problems. We studied non-verbal cognitive abilities, including non-verbal memory and attention skills, in order to evaluate the impact of primary headache on these domains. The latest version of the cognitive battery Leiter International Performance Scale – Third Edition (Leiter-3), a non-verbal test, was administered to 35 children and adolescents affected by migraine or tension-type headache and to 23 healthy subjects. We found that frequency of attacks and headache disability (evaluated with the Pediatric Migraine Disability Assessment Score Questionnaire) significantly correlate with non-verbal memory and sustained attention skills. However, we found that headache disability has a significant impact on specific cognitive domains related to sustained attention and non-verbal memory skills. The relationship between headache and memory/attention deficits may have an explanation based on a possible common physiopathology ground, including noradrenergic and dopaminergic pathways.

## Introduction

Headache has a significant impact on the quality of life of both affected patients and their families. This disorder is very common among children and adolescents (1). According to a recent review covering epidemiological studies published in the past 25 years, the estimated overall mean prevalence of headache among children and adolescents was 54.4% (range: 43.1–65.8), and it resulted more common in female than male subjects (2). Tension-type headache (TTH) and migraines are the most common types of primary headache in pediatric patients (3, 4). Several studies found an impairment in memory and attention abilities in adult population of migraneurs (5–7). Instead, the impact of primary headache on cognitive functions is not yet comprehensively studied in children and adolescents; in fact, studies regarding the effect of primary headache on neuropsychological functions showed variable and not consistent results. Villa et al. found deficits in selective and alternate attention in children with migraine (8). D’Andrea et al. described an impairment in both short-term and long-term memory in a sample of migraneurs children, if compared to healthy controls (9). Another study reported visuo-motor processing speed impairment in both migraneurs children with and without aura (10). In other studies, lower verbal Intelligence Quotient (IQ) in children with primary headache compared to healthy subjects (11–13) was reported. Therefore, the aim of this study was the evaluation of any deficit in non-verbal cognitive abilities, including memory and attention abilities, in children and adolescents affected by migraine and TTH. In addition, we investigated the effects of headache clinical features on cognitive functioning in children and adolescents with migraine and TTH.

## Materials and Methods

We recruited 35 patients admitted to the University of the Study of Bari “Aldo Moro”, affected by primary headache. Recruitment lasted between February and July 2017. The inclusion criteria for the study were: (a) age between 11 and 18 years; (b) diagnosis of primary headache according to the International Classification of Headache Disorders (third edition, beta version) (ICHD-3 beta version). The exclusion criteria were: presence of any anomaly reported at the neurological examination; presence in anamnesis of previous severe head trauma or epilepsy, presence of either major systemic or other neurologic or psychiatric disorders; neuroradiological abnormalities; current or previous use of medications with direct or side effects on central nervous system. All patients were assessed during the interictal period and they did not take anti-migraine medications. The control group included 23 healthy subjects, randomly recruited among children and adolescents attending local state schools. They were matched for age with patients.

For this study, an ethical review process by the Local Ethics Committee of Azienda Ospedaliero-Universitaria Policlinico di Bari (Italy) was not required, since all the procedures within the study assessment were included in the headache diagnostic protocol of our Child and Adolescence Neuropsychiatry Unit. All the participants were recruited after obtaining a written informed consent by their parents.

### Assessment

All patients underwent a general assessment including complete and detailed anamnesis, general and neurological examination, blood tests (blood cells count, liver and renal functions, B12 vitamin, folate and homocysteine dosage), brain magnetic resonance imaging (MRI) examination. Headache assessment included the administration of the Pediatric Migraine Disability Assessment Score Questionnaire (PedMIDAS) (14) and the administration of a questionnaire created *ad hoc* in order to assess the following characteristics: headache diagnosis, disease duration (expressed in years), and frequency of the attacks (number of events/month).

PedMIDAS is a questionnaire developed in order to assess migraine disability in pediatric and adolescent patients. It has been tested and validated for ages 4–18 years, and it is intended to be self-administered by the patients and their parents. It includes five questions: the first two questions are related to the impact of the headache on school performance, while the last three questions focuses on the impact of the headache on home or social activities. The score is a simple composite of the total of five questions. On the basis of this score, the disability grade of the headache is assessed in four levels: no or low disability (grade I; score: 0–10); mild disability (grade II; score: 11–30); moderate disability (grade III; score: 31–50); severe disability (grade IV; score greater than 50). All the participants underwent a cognitive assessment, including the administration of the Leiter International Performance Scale – Third Edition (Leiter-3) (15). The test is completely non-verbal, which means that neither the examiner nor the examinee is required to speak; it covers an expanded age range (3–75 + years). The Leiter-3 IQ score is not significantly influenced by the examinee’s language skills or by educational, social, or family experience. It focuses on fluid intelligence, considered by many the truest measure of a person’s innate ability. Leiter-3 includes two distinct batteries: the Cognitive scale and the Attention/Memory scale. The Cognitive Scale is composed by the following subtests: Figure Ground (FG), Classification and Analogies (CA), Sequential Order (SO), Form Completion (FC), and Matching/Repeated patterns (optional subtest); the administration of this scale requires maximum 45 min. Attention and Memory scale subtests are as follows: Attention Sustained (AS), Forward Memory (FM), Reverse Memory (RM), Attention Divided (AD), Non-verbal Stroop Congruent (NScc), and Non-verbal Stroop Incongruent (NSic). The non-verbal Stroop effect (NSeff) is the result of the difference between the correct answers of Nsic and NScc. It is an index that allows to evaluate the examinee’s ability to keep the attention on a precise stimulus in the presence of a distracter element. The administration requires about 30 min. The cognitive scale provides a global non-verbal IQ (nvIQ), obtained by the conversion of the sum of four subtest-scaled scores, preferentially FG, FC, CA, and SO. The Attention and Memory Scale (A/M) provides two composite scores: the first one is the Non-verbal Memory score (nvM), obtained by the sum of subtest-scaled score of FM and RM; the second is the Processing Speed score (PS), derived by the sum of the subtest-scaled score of AS and NSic (number correct). For each subtest, the raw score is the sum of the correct responses marked on the Record Form; raw scores of each subtest are converted into normalized scaled scores, using standard norm tables for the age range that includes the examinee chronological age.

### Statistical Analysis

A descriptive analysis of demographic and clinical data was conducted for both patients and control group. Age and gender between the two groups were compared using, respectively, the non-parametric test Mann–Whitney U and the Fischer’s exact test. The Mann-Whitney U test was also used to analyze the difference of Leiter-3 scores between patients and control groups. In addition, in the patients group, we conducted a correlation analysis in order to evaluate the relationship between the duration of the disease and the frequency of the attacks with Leiter-3 scores. Spearman’s rho (r) was used to describe the strength and direction of relationship between these variables.

Moreover, independent variables (severity, duration, and frequency of headache) were analyzed in the multiple regression analysis in order to evaluate the impact of headache clinical features on cognitive performances (Leiter-3 subtests) in our sample. All the statistical analyses were considered significant with a *p*-value equal or lower than 0.05. For statistical processing, we used the Statistical Package for Social Science version 20.0.

## Results

Demographical data (age and sex distribution) of both groups, diagnosis description, and main characteristics of the headache (migraine with/without aura, TTH; disease duration; frequency of attacks) are reported in Table 1. Patients and control group were age and sex matched. Leiter-3 scores global comparison between patients and control group are summarized in Table 2. Nobody showed a global cognitive impairment, non-verbal-IQ mean value in patients group was 97.6 ± 11.3. No statistical significant differences were found in the comparison of Leiter-3 scores between patients and control group. Correlation analysis between disease duration and Leiter-3 scores did not show statistical significant results. Instead, a significant negative correlation was found between the frequency of attacks and nvM (*r*: −0.434; *p* < 0.05) and RM (*r*: −0.393; *p* < 0.05) (Table 3). Moreover, we found a significant negative correlation between PedMIDAS scores and some Leiter-3 subtest scores: FG (*r*: −0.385; *p* < 0.05), AS (*r*: −0.354; *p* < 0.05), Nsic (*r*: −0.375; *p* < 0.05), NScc (*r*: −0.467; *p* < 0.05), PS (*r*: −0.341; *p* < 0.05) (Table 3).

**Table 1:** Demographic and clinical data of patients and control group.

	Patients		Controls		*p*-Value
Number	35		23		
Sex (female)	15		9		>0.05
Sex (male)	20		14		>0.05
Migraine with aura	2		–		–
Migraine without aura	24		–		–
Tension-type headache	9		–		–

	**Mean**	**SD**	**Mean**	**SD**	

Age (years)	14.89	3.2	14.75	3.17	>0.05
Disease duration	4.49	3.1	–	–	–
Frequency of attacks (number of events/month)	6.14	6.4	–	–	–

**Table 2:** Global comparison of Leiter-3 scores between patients and control group.

	Subtest-scaled score, mean (SD)		*p*-Value
	Patients (*n* = 35)	Controls (*n* = 23)	
FG	9.37 (3.0)	8.43 (3.0)	0.260
FC	9.20 (2.7)	9.09 (2.3)	0.860
CA	10.09 (2.7)	9.70 (3.1)	0.873
SO	9.97 (2.6)	10.22 (2.1)	0.619
nvIQ	98.03 (13.4)	95.83 (10.5)	0.679
AS	8.97 (2.4)	10.00 (3.0)	0.331
FM	9.29 (3.1)	9.39 (2.4)	0.968
RM	9.06 (2.7)	9.36 (2.3)	0.591
Nsic	10.31 (3.1)	9.87 (2.1)	0.592
NScc	9.71 (3.2)	9.39 (3.0)	0.829
NSeff	10.00 (3.2)	8.78 (3.3)	0.135
nvM	94.06 (17.6)	93.52 (21.7)	0.644
PS	96.23 (16.3)	100.17 (14.4)	0.435

*FG, Figure Ground; FC, Form Completion; CA, Classification/Analogies; SO, Sequential Order; nvIQ, non-verbal Intelligence Quotient; AS, Attention Sustained; FM, Forward Memory; RM, Reverse Memory; Nsic, Non-verbal Stroop Incongruent; NScc, Non-verbal Stroop Congruent; NSeff, Non-verbal Stroop Effect; nvM, non-verbal Memory; PS, Processing Speed*.

**Table 3:** Correlation analysis between Leiter-3 scores and frequency of the attacks, duration of disease and PedMIDAS scores in patients group.

		FG	FC	CA	SO	nvIQ	AS	FM	RM	Nsic	NScc	NSeff	nvM	PS
Frequency of attacks (no of events/month)	*r*	−0.253	−0.065	−0.154	0.029	−0.166	−0.243	−0.317	−0.393	−0.181	−0.118	−0.036	−0.434	−0.253
	*p*	0.142	0.712	0.378	0.869	0.341	0.160	0.063	0.020*	0.299	0.500	0.839	0.009*	0.142

Duration of disease	*r*	−0.121	0.162	−0.150	0.251	0.008	−0.161	−0.119	0.004	−0.234	−0.191	−0.138	0.015	−0.110
	*p*	0.487	0.354	0.391	0.146	0.964	0.355	0.497	0.982	0.176	0.272	0.428	0.931	0.530

PedMIDAS scores	*r*	−0.385	−0.095	−0.162	−0.158	−0.260	−0.354	−0.288	−0.260	−0.375	−0.467	0.121	−0.286	−0.341
	*p*	0.023*	0.589	0.352	0.366	0.131	0.037*	0.093	0.131	0.027*	0.005*	0.488	0.095	0.045*

FG, Figure Ground; FC, Form Completion; CA, Classification/Analogies; SO, Sequential Order; nvIQ, non-verbal Intelligence Quotient; AS, Attention Sustained; FM, Forward Memory; RM, Reverse Memory; Nsic, Non-verbal Stroop Incongruent; NScc, Non-verbal Stroop Congruent; NSeff, Non-verbal Stroop Effect; nvM, non-verbal Memory; PS, Processing Speed.

*p < 0.05.

The multivariate regression analysis (Table 4) showed that only PedMIDAS score was significantly associated with FG (95% CI; *p* = 0.04), FM (95% CI; *p* = 0.04), NSeff (95% CI; *p* = 0.01) scores; on the contrary, we did not found a significant association of PedMIDAS score on FC (95% CI; *p* = 0.5), CA (95% CI; *p* = 0.51), SO (95% CI; *p* = 0.67), nvIQ (95% CI; *p* = 0.19), AS (95% CI; *p* = 0.13), RM (95% CI; *p* = 0.22), NSic (95% CI; *p* = 0.07), NScc (95% CI; *p* = 0.06), PS (95% CI; *p* = 0.14), and nvM (95% CI; *p* = 0.24). We did not found a significant association of frequency of attacks and disease duration with Leiter-3 subtests scores (Table 4).

**Table 4:** Multivariate regression analysis between PedMIDAS scores, disease duration and frequency of attacks and Leiter-3 subtests scores.

					95% CI	
		*B*	*t*	*p*-Value	Inf	sup
	FG					
PedMIDAS		−0.91	−2.13	0.04*	−1.78	−0.04
Disease duration		−0.09	−0.54	0.60	−0.42	0.24
Frequency of attacks		−0.07	−0.96	0.34	−0.23	0.08
	FC					
PedMIDAS		−0.28	−0.66	0.50	−1.13	0.57
Disease duration		0.15	0.93	0.36	−0.18	0.47
Frequency of attacks		−0.06	−0.74	0.47	−0.21	0.10
	CA					
PedMIDAS		−0.28	−0.67	0.51	−1.13	0.57
Disease duration		0.15	0.93	0.36	−0.18	0.47
Frequency of attacks		−0.06	−0.74	0.47	−0.21	0.10
	SO					
PedMIDAS		−0.18	−0.43	0.67	−1.04	0.68
Disease duration		−0.14	−0.85	0.40	−0.46	0.19
Frequency of attacks		−0.03	−0.43	0.67	−0.19	0.12
	nvIQ					
PedMIDAS		−0.52	−1.35	0.19	−1.31	0.27
Disease duration		0.24	1.64	0.11	−0.06	0.54
Frequency of attacks		0.05	0.78	0.44	−0.09	0.20
	AS					
PedMIDAS		−3.16	−1.57	0.13	−7.27	0.95
Disease duration		0.27	0.36	0.72	−1.29	1.83
Frequency of attacks		−0.16	−0.44	0.66	−0.90	0.58
	FM					
PedMIDAS		−0.73	−2.11	0.04*	−1.43	−0.03
Disease duration		−0.05	−0.38	0.70	−0.32	0.22
Frequency of attacks		−0.06	−0.92	0.37	−0.18	0.07
	RM					
PedMIDAS		−0.59	−1.27	0.22	−1.53	0.36
Disease duration		−0.20	−1.12	0.27	−0.56	0.16
Frequency of attacks		−0.07	−0.90	0.38	−0.24	0.10
	Nsic					
PedMIDAS		−0.73	−1.89	0.07	−1.52	0.06
Disease duration		0.01	0.05	0.96	−0.29	0.31
Frequency of attacks		−0.10	−1.44	0.16	−0.24	0.04
	NScc					
PedMIDAS		−0.89	−1.95	0.06	−1.82	0.04
Disease duration		−0.11	−0.61	0.55	−0.46	0.25
Frequency of attacks		−0.04	−0.45	0.66	−0.20	0.13
	Nseff					
PedMIDAS		−1.17	−2.74	0.01*	−2.04	−0.30
Disease duration		−0.09	−0.55	0.59	−0.42	0.24
Frequency of attacks		0.15	2.00	0.05	0.00	0.31
	PS					
PedMIDAS		−3.81	−1.50	0.14	−9.00	1.38
Disease duration		−0.32	−0.33	0.74	−2.29	1.65
Frequency of attacks		−0.78	−1.71	0.10	−1.71	0.15
	nvM					
PedMIDAS		0.57	1.20	0.24	−0.40	1.54
Disease duration		−0.29	−1.58	0.12	−0.65	0.08
Frequency of attacks		−0.02	−0.24	0.81	−0.19	0.15
						

*FG, Figure Ground; FC, Form Completion; CA, Classification/Analogies; SO, Sequential Order; nvIQ, non-verbal Intelligence Quotient; AS, Attention Sustained; FM, Forward Memory; RM, Reverse Memory; Nsic, Non-verbal Stroop Incongruent; NScc, Non-verbal Stroop Congruent; NSeff, Non-verbal Stroop Effect; nvM, non-verbal Memory; PS, Processing Speed; CI, Confidence Interval*.

*p < 0.05.

## Discussion

The impact of primary headache on the cognitive functions in the pediatric population is not yet comprehensively understood. For the first time, in 1989, D’Andrea et al. described a specific impairment in short- and long-term memory in a sample of 20 migraneurs children using “The logical memory test” and the “Rey memory test” (9). On the other hand, Haverkamp et al. did not find significant differences comparing processing speed performances of migraneurs with healthy controls (16). Later, in an Italian cross-sectional study, Parisi et al. administered the Wechsler Intelligence Scale for Children-Revised to 82 children affected by migraine without aura and TTH (11). They found significant worse performances in terms of total IQ (TIQ) and verbal skills, comparing the subgroup of patients with healthy controls. Moreover, they found a negative correlation between TIQ, verbal IQ, and performance IQ and the frequency of the attacks. Lastly, they found a significant correlation between the age of headache onset and the TIQ. Therefore, these results suggested a possible relationship between the severity of the headache and the global cognitive abilities. In addition, Esposito et al. found lower verbal performance in patients with TTH if compared to migraneurs patients and healthy controls, using the Wechsler Intelligence Scale for Children-third edition (WISC-III) (13). In these previous studies, the researchers investigated cognitive competences using protocols requiring verbal skills. We studied non-verbal cognitive abilities, such as fluid intelligence, non-verbal memory, attention, in children and adolescents affected by primary headache, using the latest version of the cognitive battery Leiter-3 (15). This is a non-verbal test allowing the evaluation of those skills, without significant bias determined by the cultural of individual, educational, and social factors. Differently from the previous edition, the Leiter-3 cognitive battery provides a non-verbal IQ with just four subtests. The internal structure of this test allows to study non-verbal attention and memory skills separately from other cognitive aspects. In accordance to previous studies (10, 13, 16), primary headache has not an impact on global non-verbal cognitive abilities. We found that frequency of attacks and headache disability (evaluated with the PedMIDAS) significantly correlate with non-verbal memory (nvM and RM) and sustained attention skills (FG, AS, Nsic, NScc, PS). However, we found that headache disability has a significant impact on specific cognitive domains related to both non-verbal memory and sustained attention skills (FG, FM, NSeff). Non-verbal Memory is obtained by the sum of RM and FM subtest-scaled scores. RM subtest measures the memory span of figures backwards. Reverse memory is a complex mental activity that requires storing and manipulating information; it implies a good working memory, not required for the forward memory subtest. FG subtest is a task of simple visual interference, similar to a visual recognition but with distracters and enlargements. In this subtest, the performance is related to the individual cognitive flexibility (i.e., his ability to shift attention), since he needs to shift attention from a well-defined figure to a complex background, modifying in a sense his perceptual set (17, 18). AS subtest requires attention and selectivity skills; the examinee is asked to perform a simple repetitive task that does not require any new cognitive processing. An individual who shows deficiency in this subtest may have an underlying attentive problem. PS score depends on the performance in AS and Nsic subtests; hence, it is considered a measure of attention, working memory, and inhibitory skills of the examinee. Therefore, our results suggest that a higher headache disability may have an impact on non-verbal memory, attention sustained. Le Pira et al. found both short-term and long-term memory defects in 30 adult migraneurs, when compared with 14 healthy subjects. The authors suggested that this impairment was probably related to defective learning strategies and memory recall mechanisms (6). In 2002, Calandre et al. revealed disturbances in memory, attention, and visuo-motor speed processing among adult migraneurs experiencing higher frequency of attacks. Moreover, they found a potential relationship between brain perfusion abnormalities and some cognitive deficits, such as visual and verbal memory skills (5). Later, Villa et al. administered a visual attention assessment to 30 migraineurs children and then they compared their performance with a control group. For this assessment, the authors used three tests: Trail Making Test (TMT) A/B, Letter Cancelation Test and the Brazilian computerized Visual Attention Test. The migraine group showed worse results in selective and alternate attention tasks, when compared with the control group (8). Later, Moutran et al. compared 30 children migraneurs with 30 healthy children; both groups underwent a cognitive assessment using WISC-III. All the participants had a normal cognitive performance, but children with migraine and TTH showed worse performance in attention, memory, and speed processing information (12). The relationship between headache and memory/attention deficits may have an explanation based on a possible common physiopathology ground. These cognitive functions involve several brain regions, both cortical and subcortical areas (5, 11). Moreover, some neurotransmitters, such as dopamine and noradrenaline, mediate an important role in attention, working memory, and learning tasks (19–21). Both dopaminergic and noradrenergic pathways are also involved in migraine pathogenesis (22–25). In fact, noradrenaline modulates hyperexcitability of the trigeminovascular system; low levels of this mediator predispose migraneurs to more intense and long lasting cardiovascular symptoms, such as tachycardia and high pressure levels, associated with the migraine attack (23, 26). On the other hand, normal levels of noradrenaline promote adequate levels of selective, alternate and also sustained attention, mediating cortex, and subcortical pathways via-α2 noradrenergic receptors (27). Dopaminergic connections modulate not only trigeminovascular system excitability but also cognitive functions involving working memory and attention skills (20, 23, 24), mediating prefrontal cortex and thalamo-cortical pathways (20, 28). Eventually, these common neurobiological aspects may explain the occurrence of memory/attention deficits and headache disorder.

In conclusion, the results of this study revealed that migraine and TTH may have an impact on both non-verbal memory and attention skills in children and adolescents. The findings confirm the importance of a cognitive assessment in these patients, especially regarding memory and attention skills, also for the impact of these abilities on academic performances. Moreover, any possible cognitive impairment, along with the severity of the disease, should be considered in anticipation of any prophylactic pharmacological therapy.

However, the small size of the sample was a limitation of this study; eventually, further studies with larger samples could better define the relationship between headache and its impact on non-verbal cognitive abilities. Moreover, it would be interesting to follow the evolution of these aspects over time, in order to evaluate how much they can affect the prognosis of the primary headache with an onset during developmental age if compared to the disorder with an onset during adulthood.

## Ethics Statement

For this study, an ethical review process by the Local Ethics Committee of Azienda Ospedaliero-Universitaria Policlinico di Bari (Italy) was not required, since all the procedures within the study assessment are included in the headache diagnostic protocol of our Child and Adolescence Neuropsychiatry Unit. All the participants were recruited after obtaining a written informed consent by their parents.

## Author Contributions

LM, MB, and PL contributed to the conception, the design, the supervision, and the final approval of the work. RP, MS, MM, and SS contributed to the enrollment of the participants, to the general and cognitive assessment and to data collection. FC performed the statistical analysis. All authors reviewed and edited the manuscript.

## Conflict of Interest Statement

The authors declare that the research was conducted in the absence of any commercial or financial relationships that could be construed as a potential conflict of interest.
